# An organic jelly made fractal logic gate with an infinite truth table

**DOI:** 10.1038/srep11265

**Published:** 2015-06-18

**Authors:** Subrata Ghosh, Daisuke Fujita, Anirban Bandyopadhyay

**Affiliations:** 1 National Institute for Materials Science (NIMS), Advanced Key Technologies Division, 1-2-1 Sengen, Tsukuba, Japan.; 2 Mass General Hospital (Harvard Medical School), Building-114, 16th Street, Charlestown, Boston, Massachusetts-02129 USA

## Abstract

Widely varying logic gates invented over a century are all finite. As data deluge problem looms large on the information processing and communication industry, the thrust to explore radical concepts is increasing rapidly. Here, we design and synthesis a molecule, wherein, the input energy transmits in a cycle inside the molecular system, just like an oscillator, then, we use the molecule to make a jelly that acts as chain of oscillators with a fractal like resonance band. Hence, with the increasing detection resolution, in the vacant space between two energy levels of a given resonance band, a new band appears, due to fractal nature, generation of newer energy levels never stops. This is natural property of a linear chain oscillator. As we correlate each energy level of the resonance band of organic jelly, as a function of pH and density of the jelly, we realize a logic gate, whose truth table is finite, but if we zoom any small part, a new truth table appears. In principle, zooming of truth table would continue forever. Thus, we invent a new class of infinite logic gate for the first time.

Laser etching that draws millions of logic gates on Silicon would soon cease to shrinking, far below the computation limit^1^. All routes to stretch beyond, like, processing & memorizing in a single device^2^,^3^,^4^, non-physical wiring^5^,^6^,^7^ follow the same principle, —without reducing the device size, more information cannot be packed & processed in a fixed space (it gravitates to Moor’s law). “Infinite logic” principle^8^,^9^,^10^,^11^ is just the opposite, if realized, it would replace “bits” with continuum that is critically demanded for a true adaptive logic^12^, and often seen as a prerogative of chemical computing^13^,^14^,^15^. Though fractals promise to complement the technological demands for a true “Infinite logic”^16^,^17^,^18^, there exists no clear evidence though the hunt has peaked in the bio-systems^19^,^20^,^21^,^22^,^23^. In dielectric physics, it has been theoretically shown that in a chain of linear oscillators, the system develops fractal distribution of energy levels^24^. It means, just like Mandelbrot fractal, if one zooms a part of the resonance band, a new band appears. Here, we use this principle to design and synthesis a new material that shows the similar property, and realize the infinite truth table. This makes the trend to continuous miniaturization irrelevant, unprecedented technologies pitched with the continuum hypothesis since the 1870 s^8^,^9^,^10^,^11^,^25^, like infinite packing density, universal programmable matter^26^ and a time resolution beyond any measurable machine^27^, would henceforth continue to transfer equations into devices.

All machines we see around are made of a finite state logic (0 and 1), it’s a historical irony that an infinite state logic^8^,^9^,^10^,^11^ (110001010111… to infinity) was born much before^25^ the finite logic. Since we failed to create an infinite state in a finite machine, the promises of incredible technologies remained in the equations, never seen the lab light. Parameters governing the nature are made of infinite series, triggering the quest to find deterministic solution in random or chaotic chemical systems inspired by living machines and in synthetic chem-bio fusion systems, both issues largely dominated the logic gate literatures^19^,^20^,^21^,^22^,^23^. Moreover, the literatures are rich in interpreting complex biological events as logic gate to learn the decision making process of nature in simple terms, however, what that is lost in simultaneity could never be recovered in sequential discretized finite state machines^28^. Thus, we need basic computing elements that can store & process an infinite series. Failing to realize such device has put thrust on chaos, where, knowing the input generates the output irrespective of complexity. In the Artificial intelligence models, the determinism is ensured by manipulating randomness, for subtle advantages^29^. All adventures on randomness, chaos and determinism have finite logic skeleton (**chaos and determinism online text A**), in contrast, for an infinite logic, the resolution of solution continuously increases towards the exact number, but zoom-in never ceases. Thus, incompleteness argument of Godel^30^ translates into an infinite-series that computing element to be made would demonstrate. Except fractal, no other engineering concept is as close as such quasi-determinism^23^, hence, in this solitary adventure, creating such a fractal in an organic molecular system has been the primary goal.

Theoretical calculations (see Methods) suggest that a linear chain of oscillators generate a fractal distribution of energy levels. Therefore, we need an electromagnetic molecular oscillator potentially forming a chain. For periodic oscillation, we need a minimum of three energy-trapping centers in a molecule. Since covalent bonding dilutes pristine molecular property hence it’s a critical challenge to synthesize a weak bonded structure with three dopants in a suitable matrix. We use dendrimer matrix^31^ to harness its fractal like energy transmission. Then, we add a pH sensor molecule, a molecular rotor whose rotation could be tuned by varying density (M, see Movie 1 and Movie 2 for molecular rotor dynamics in theory and in STM) and dope a multi-level electromagnetic switch^32^,^33^ inside the dendritic cavity to create a triangular energy transmission pathway. The dendritic derivative is then transformed into a jelly to mimic a linear chain feature, wherein simply by varying pH and density one could manipulate the boundary values of its linear chain oscillation. Thus, an infinite truth table is experimentally realized (**the difference between fractal and conventional logic gates:** Table 1 **online**), a demand is met for infinite computer^34^.

Fig. 1a shows a generic design of a dendritic nano-platform. The most important step to make a generic programmable oscillator is to select a fit matrix. Figure. 1b shows geometries of a dendrimer branches. Lesser is the conjugation higher is the mobility of branches. Complete conjugation makes the structure planar, restricting the interplanar energy exchange. Partial conjugation balances the two (**molecular dynamics of distinctly conjugated branches in** movie 3 **online**). The surface-attached functional groups of dendrimer, restricts the randomness of its branch-dynamics. Functionalization of semi-conjugated branches block the inter branch energy transfer, hence for an effective triangular energy transmission path, completely non-conjugated PAMAM matrix is found suitable.

Figure. 1c shows synthesis protocol for 5th generation PAMAM molecule or P. First, Nile Red molecule (C) is doped inside to get PC, connect 4 NIR 797 pH sensors S and then 32 molecular rotors M are connected (M is 2-Methoxy-phenyl ethynyl)-naphthalen-1-ylamine designed and synthesized by us), we get PCMS (see Methods and supporting information online). Figure. 1d shows that a linear chain of PCMS generates fractal resonance band where infinite number of discrete yet theoretically predictable energy levels reside (see methods for calculation).

In order to confirm that PCMS molecule is the desired electromagnetic oscillator, we need experimental and theoretical evidence that say, if an input energy would first be captured by S, then C and then M, then this S → C → M path is universal, all routes except this are blocked. In the Combined Excitation Emission Spectroscopy (CEES, see Methods, online text B), there are distinct peaks for P, C, M and S in the CEES plot. Therefore, if we change pH or density gradually, we find that the high intensity peaks in the CEES plot move along a line as shown in the Fig. 2a–f, wherein, we see that the peaks for S, C and M turns bright sequentially. We demonstrate the triangular energy transmission path in Movie 4 online, wherein one can see that M and S exchanges energy only via C (Fig. 2g).

Theoretically, we have tracked the energy transmission path by mimicking pH and density variation by putting PCMS in a potential box and increasing the number of protons and PCMS in that box. As the electromagnetic potential (blue negative and red positive) changes in PCMS (Fig. 2h), one can track the S → C → M path; one of the four lobes of PCMS is zoomed below, we can see the discrete, isolated red and blue regions. We have plotted the discrete energy levels for PCMS, P, C, M and S pristinely and then have shown how fractal distribution of energy level appears as a function of pH and density in the Movie 5 online. One of the most remarkable feature that the reader would notice in Movie 5 is that the fractal energy level distribution for pH and density are out of phase, this is a classic textbook like condition to build a simple harmonic oscillator. Thus, PCMS in solution forms the linear chain of oscillators, as pH and density tunes photo-induced resonance^35^,^36^.

Moreover, the regularity in intensity variation in Fig. 2h as a function of density (above) and pH (bottom), establishes the foundation of designing a logic gate. Therein, the peaks move along a line, the change is not random. Commercially available 14 low-resolution sensors can detect 14 excitation-emission points along the line in Fig. 3a (from the beaker). Wehave varied pH and density and created a database of CEES plots (**online pH-density CEES database, separate pdf file**). Say, we move along the depicted line in Fig. 3a, we find four major regions where peaks move (Fig. 3b). If we have a 0.5 eV resolution sensor, then left to write in the four regions we could detect 1 + 5 + 6 + 2 = 14 movement steps along the line. From this line, we select three output points for creating a truth table. For simplicity, we construct a truth table for “two inputs, three outputs” logic gate, the outputs could be much more. Only 10 output points are shown in eV in the table Fig. 3c. Keeping the pH constant, we have changed the density and observed the fluorescence intensity variation at different sensory outputs. Figure. 3c table shows that for 0 to 4, five different decisions could be taken. Since we could take any small region among four in Fig. 3b, zoom that region just by increasing the sensor resolution and get distinct outputs. Keithley 6430 with pre-amp has 10 × 10^−18^ eV sensors, hence starting from a 2 × 3 logic gate with 10 values we can zoom any pH or density region to harness at least 0.5 to 10^−5^ eV region, i.e. we can zoom the same truth table 10^6^ times at the least, and hence it is a fractal logic gate (**the fractal logic gate by CEES** Movie 5 **online**). The infinite state accessibility depends on the efficiency of measuring system.

Semi-empirical computation and the experiment suggest that the peaks for path M ↔ C ↔ S in the CEES change non-linearly as a function of pH and density. Peaks, shift differently in different regions, it enables user to harness local energy states and applying allowed/restricted transition rules. The PCMS molecules on the Si/SiO_2_ surface under SEM start spontaneous motions as shown in Fig. 4a (**high resolution single PCMS molecule oscillation in** Movie 6 **online**). Starting patterns and schematic paths for three rows show that a specific path is adopted in spite of induced noise. If the number of PCMS is increased, the change in the input condition is spontaneously reflected in the changed path dynamics, —the system is adaptive. Most interestingly, second and third rows in Fig. 4a show that the adopted path changes with the input arrangement, though the number of PCMS remains the same. This resembles chaotic computing, the dynamics is encoded in the input arrangement, knowing the input we can get the output. Figure. 4b shows that if we continue to increase PCMS, the adopted random motions, try to follow a well-defined geometric path, the time-cycle is predominant. This is important as quasi-determinism generates chaos and deterministic choices, we have more generic computing element in operation. Also, readers could see the linear chain of PCMS oscillators live in Fig. 4a, which is the foundation of the fractal resonance band. One could use this oscillator for treating cancer and Alzheimers^37^.

Period, or time cycle or rhythm remains constant even under random PCMS motions. An energy transmission route (M ↔ C ↔ S) is a rhythm encoded in the atomic arrangements that PCMS restores under noise, —a key to its adaptive behavior. It allows PCMS to sustain a defined geometric path on the surface under noise and a logical fluorescence output in solution even when pH and density range are changed continuously, testing the system’s high resolution withstanding capability. Unprecedented resolution embeds a unique feature, memory; zooming to expand any part of the operational matrix converting say, a 10 × 10 pH-density matrix into a 1000 × 1000 one, thus, a nested network holds astronomically large data^38^. Even for a large matrix, time to search is determined by the time cycle of the smallest matrix, this is what we call “instantaneous decision-making” —for an observer sitting on the lowest resolution world (Table 1 online)^38^.

## Methods summary

### Theory, how a molecular oscillator generates fractal energy band: creation of infinite energy levels

**Figure 1:**
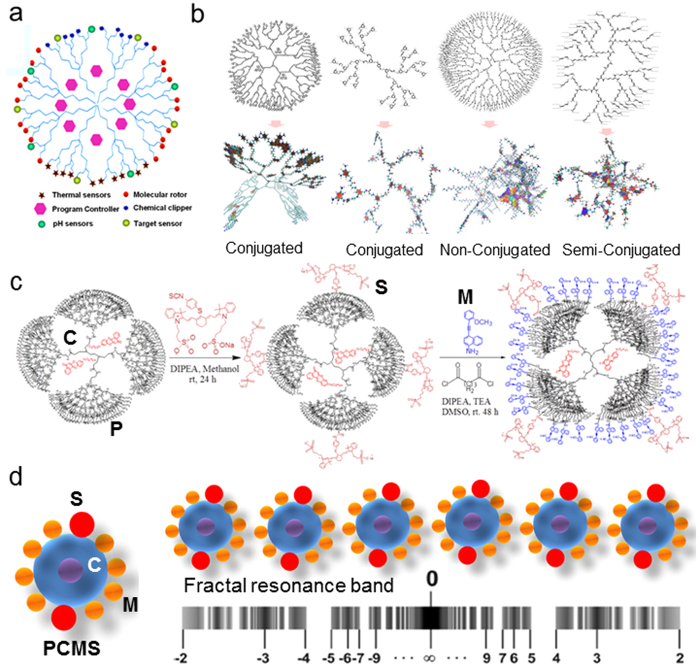
(**a**) A generic design for dendrimer based complex multi-functional derivative.(**b**). **Left to right 1,2,3,4** Molecular structures (above) and their energy minimized structures (below) are shown here for **1, 2** and **3**. **4.** First, **1 & 2.** Sp^2^ hybridized conjugated dendrimer (no encapsulation is possible) **3** 5th Generation PAMAM dendrimer (non-conjugated, Sp^3^ hybridized) **4** Design of a semi conjugated dendrimer structure created by a linear combination of Sp^2^ and Sp^3^ hybridized states. For all panels, the potentially active planes taking part in the charge transfer across the molecular structure are shaded. (**c**) In the first step reaction scheme for doping two Nile red molecules C is demonstrated (MALDI-TOF mass spectroscopy study confirms the number), then, four sensor molecules (S = NIR797 isothiocyanate) are attached and in the third step 32 molecular machines are bonded with the PAMAM surface. (**d**) Linear chain of PCMS and fractal/infinite resonance band (see methods).

If we synthesize a molecular oscillator with a single resonance frequency f_0_, the oscillator will have a higher mode oscillation frequency f_1_ and the relation between them f_1_/f_0_ = n. Now for a nested waveform network, say one waveform encapsulates 3 waveforms in it, and that continues, then first we get f_1_ = f_0_, then f_1_ = 3f_0_, then f_1_ = 9f_0_, hence in general we can write f_n_,p = f_0_ n^r^. In this way, the resonant frequency spectrum due to one particular symmetry can be represented as a logarithmic fractal spectrum. We can clearly see that a singular waveform fraction continues to occur in the chain of oscillators. If f_0_ is fundamental resonance of one oscillator and f is frequency of the chain then using simple expression of continued fraction we get the resonance spectrum or a distribution of natural resonance frequencies, f = f_0_exp(S), S = n_0_/z + z/(n_1_ + z/(n_2_ + z/(n_3_+….+z/n_i_))). Now, the band we get for i = 1, is similar to band we get for i = 2 and so on, so it is a fractal, the spectrum looks like a hyperbolic function. Figure. 1d shows band for f_1_/f_0_ = 3.

### Synthesis of molecular rotor & four supramolecular architectures: Molecular Machine (MM or M) synthesis

The reactive amine group of 1-amino-4-bromonaphthalene is first protected with di-tertiary-butyl dicarbonate (Boc) to facilitate the Sonogashira coupling with 2-ethynyl anisole. In the presence of palladium acetate as catalyst and triphenyl phosphene as co-catalyst, Boc-derivative of 1-amino-4-bromonaphthalene is coupled with 2-ethynyl anisole to produce Boc-derivative of 4-(2-Methoxy-phenyl ethynyl)-naphthalen-1-ylamine, which on treatment with hydrated tetrabutyl ammonium fluoride resulted in 4-(2-Methoxy-phenyl ethynyl)-naphthalen-1-ylamine (MM). Summary of molecular dynamics simulation, spectroscopic confirmation of rotor ability is in the supporting online text A and synthesis details is in the online text B (see Figures S1 to S9 online).

### PC, PM, PCM, PCS, PCMS synthesis

To synthesize PR, PCM and PCMS, —first, PAMAM G5 dendrimer (1:2) aqueous sodium carbonate, methanol solution is taken at >9.5 pH, in presence of Nile-red dye molecules, to encapsulate two of them into the deep core of the four dendritic cavities, we get [PAMAM5-NR] dendritic box (PC, step I). Then, the PC is taken into a 10% and 40% mixture of dimethyl sulfoxide (DMSO) and acetonitrile, the sensor (NIR797isothiocyanate, S) solution in the borate buffer (50% of total volume) is added in one portion and the reaction is continued at room temperature. The primary amine groups at the PAMAM surface is then connected with the NIR797isothiocyanate dye molecule, the reaction product [PAMAM-NR]-NIR797isothiocyanate (PCS, step II) is taken to the next step. A multi-component mixture of product PCS, MM, di-isopropyl amine and triethyl amine is dissolved in dry dimethyl sulfoxide, and glutaryl chloride is added slowly to the mixture at below 20C. The reaction is carried out at room temperature for 48 h and the final product is collected, [PAMAM-NLR]-NIR797isothiocyanate-MM (PCMS, step III). For PMS, the step I is avoided, the rest remains the same; for PCM, instead of S, M attachment part is carried out on PC. In all steps the products are purified through extensive dialysis, MALDI-TOF, Raman, FTIR, NMR and step-by-step CEES spectroscopy were carried out to confirm the product nature (see Figures S1 to S9 online). The synthesis of previously reported PCM and herein described PCMS are fundamentally different (see details online).

### Combined excitation emission spectroscopy (CEES)

Combined excitation emission spectroscopy (CEES): ~200 emission spectra are recorded at excitation wavelengths with 5 nm intervals. The output intensities are plotted as a function of excitation and emission wavelengths, converted into energies (eV). From iso-contour plot, we detect peaks; at each peak, we get three values, excitation energy (Ex), emission energy (Em) and depending on the negative or positive sign of ΔE ( = Ex - Em), absorbed or emitted energy by the molecular structure during the emission process, using solution Raman & molecular dynamics we find which atomic groups use ΔE. Using this concept we evaluate band transitions for every single event, neglecting regions above Raman ridge at 45^0^ (since Ex < Em), around 45^0^, ΔE~0, there is no absorption, entire applied energy emits out. In the CEES, S peak (1.9 eV, 4.5 eV) is hidden in all CEES plots in this paper as its intensity is low compared to P, C and M.

### Theoretical simulation of PCMS energy transmission path

Theoretical simulation to track the energy transmission path was carried out by semi-empirical technique AM1, on MOPAC. To emulate the density effect, we tested 60 different conformations and estimated path variation for stress on PCMS. For pH study, we used Hyperchem 10.0, wherein we surrounded PCMS by ions as a potential box problem. Relative potential distribution as a function of proton (H+) density was studied to emulate a pH variation experiment.

## Additional Information

**How to cite this article**: Ghosh, S. *et al.* An organic jelly made fractal logic gate with an infinite truth table. *Sci. Rep.* **5**, 11265; doi: 10.1038/srep11265 (2015).

## Supplementary Material

Supplementary Data

Supplementary Movie 1

Supplementary Movie 2

Supplementary Movie 3

Supplementary Movie 4

Supplementary Movie 5

Supplementary Movie 6

Supplementary online material

## Figures and Tables

**Figure 2:**
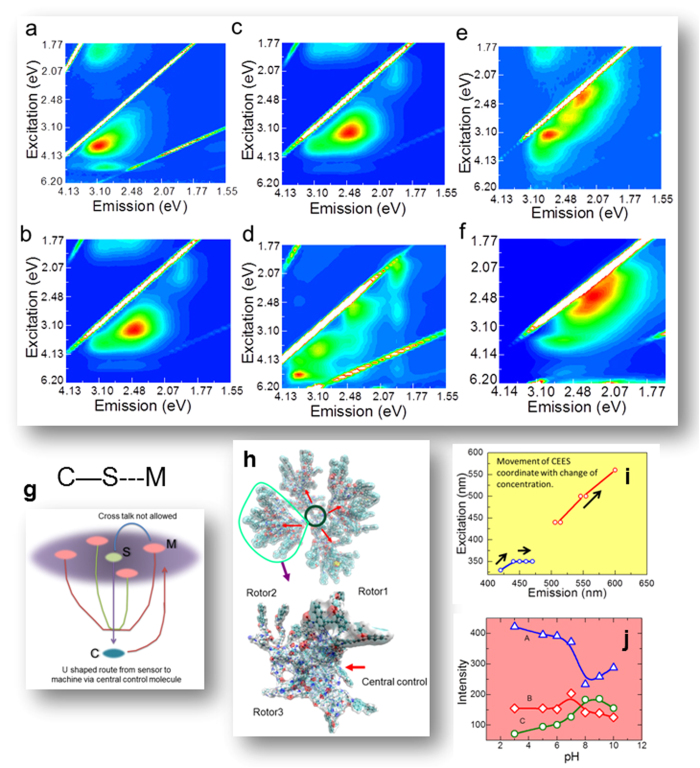
(**a-f**) (**a**) CEES spectrum for the PAMAM; (**b**). CEES spectrum for the NR-PAMAM; (**c**). CEES spectrum for NR-PAMAM; (**d**). CEES spectrum for PAMAM-NR-NIR797; (**e**). CEES for PAMAM-NR-MM; (**f**). CEES spectrum for PAMAM-NR-NIR797-MM. Note that these data are density and pH dependent. (**g**). Schematic shows CS and MC dual path (**h**) Potential surface of PCMS. One lobe is zoomed below. (i) Peaks variation with density from 2 mg/mL to 10mg/mL (**j**) Peaks variation from pH. 2 to PH 12.

**Figure 3:**
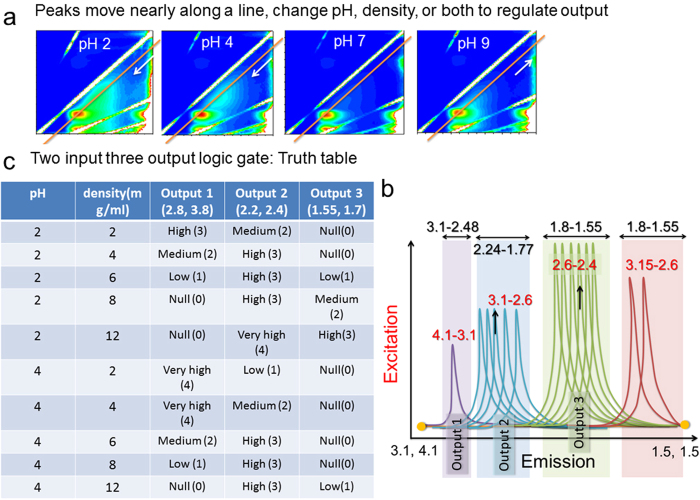
CEES, left to right pH 2, 4, 7, 9; density 2 mg/mL, a line is drawn parallel to Raman ridge where peaks move, scale same as (**a**) Fig. 2a–f. (**b**). Schematic of peaks motion paths, shows 14 locations along the line Fig. 3a (**c**) Two input three output logic gate: truth table.

**Figure 4:**
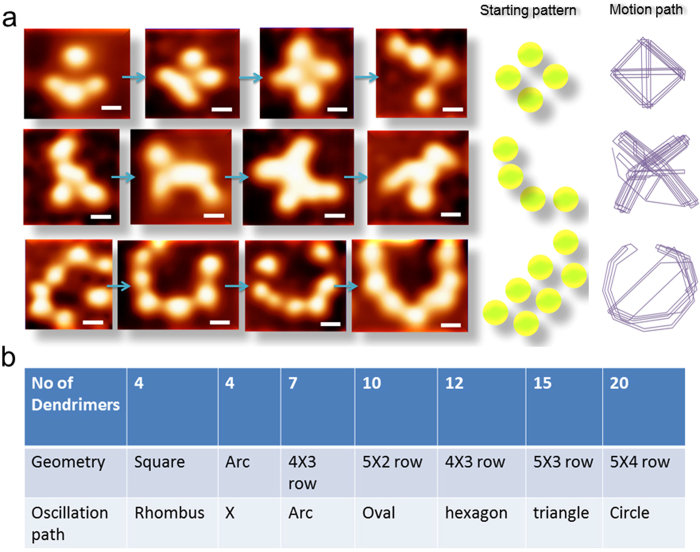
Three rows show three time profiles. First two rows Consecutive images of four swarms from left to right, time gap 20 seconds, scale bar is 8nm. **a**) The schematic of the initial arrangement is shown in the right with motion path; **Third row** Consecutive images of seven swarms from left to right, time gap 20 seconds, scale bar is 6 nm. The schematic of the initial presentation is shown in the right. (**b**). Table summarizing the geometry & oscillation geometry as function of number of PCMS.

## References

[b1] Bennett C. H. & Landauer R. The Fundamental Physical Limits of Computation. Sci. Am. 253, 48–56 (1985).2416051

[b2] Siuti P., Yazbek J. & Lu T. K. Engineering genetic circuits that compute and remember. Nat. Proto. 9, 1292–1300 (2014). 10.1038/nprot.2014.089 24810038

[b3] Bandyopadhyay A. & Acharya S. A 16-bit parallel processing on a molecular assembly. Proc. Natl. Acad. Sci. USA 105, 3668–3672 (2008). 18332437 10.1073/pnas.0703105105 PMC2268776

[b4] Biswas A. K., Atulasimha J. & Bandyopadhyay S. An error-resilient non-volatile magneto-elastic universal logic gate with ultra-low energy-delay product. Sci. Rep. 4, 7553 (2014). 25532757 10.1038/srep07553 PMC4274515

[b5] Tamsir A., Tabor J. J. & Voigt A. Robust multi-cellular computing using genetically encoded NOR gates and chemical wires. Nature 469, 212–215 (2011). 21150903 10.1038/nature09565 PMC3904220

[b6] Bandyopadhyay A., Pati R., Sahu S., Fujita D. & Peper F. A massively parallel computing on an organic molecular layer. Nature Phys. 6, 369–375 (2010).

[b7] Bandyopadhyay A., Sahu S. & Fujita D. Smallest artificial molecular neural-net for collective and emergent information processing. Appl. Phys. Lett. 95, 113702 (2009).

[b8] Kalicki J. An Undecidable Problem in the Algebra of Truth-Tables. J. Symb. Logic 19, 172–176 (1954).

[b9] Grim P., Mar G. & Denis P. S. The philosophical computer: Exploratory essays in philosophical computer modeling. [323] (MIT Press, Boston, USA, 1998).

[b10] Karp C. R. Languages with expressions of infinite length. (eds Brouwer I. E. J. *et al.* ) (North-Holland Publishing Co., Amsterdam, 1964).

[b11] Barwise K. J. Infinitary logic and admissible sets. J. Symb. Logic 34, 226–252 (1969).

[b12] Van Otterlo M. Frontiers in Artificial Intelligence and Applications, Vol 192 (eds Van Otterlo M. ) [508] (Ios Press, STM Publishing House, 2009).

[b13] Adamatzky A., Costello D. L. & Benjamin P. J. Experimental logical gates in a reaction-diffusion medium: The XOR gate and beyond. Phys. Rev. E 66, 046112 (2002). 10.1103/PhysRevE.66.046112 12443264

[b14] Lebender D. & Schneider F. W. Logical Gates Using a Nonlinear Chemical Reaction. J. Phys. Chem. 98, 7533–7537 (1994).

[b15] Steinbock O., Kettunen P. & Showalter K. Chemical Wave Logic Gates J. Phys. Chem. 100, 18970–18975 (1996).

[b16] Gentili P. L., Horvath V., Vanag V. K. & Epstein I. R. Belousov-Zhabotinsky “chemical neuron” as a binary and fuzzy logic processor. Int. J. Unconv. Comp. 8, 177–192 (2012).

[b17] Kopelman R. Fractal Reaction Kinetics, Science, 241, 1620–1625, (1988). 17820893 10.1126/science.241.4873.1620

[b18] Gordon N. L. Introducing Fractal Geometry 3rd edn [71] (Totem book 2000).

[b19] Ausländer S., Ausländer D., Müller M., Wieland M. & Fussenegger M. Programmable single-cell mammalian biocomputers. Nature 487, 123–127 (2012). 22722847 10.1038/nature11149

[b20] Sinha S. & Ditto W. L. Computing with distributed chaos. Phys. Rev. E. 59, 363–377 (1999). 10.1103/physreve.60.363 11969770

[b21] Casdagli M. Chaos and Deterministic versus Stochastic Non-linear Modelling. J. Royal Stat. Soc. Series B 54, 303–328 (1991).

[b22] Babaei M. A novel text and image encryption method based on chaos theory and DNA computing. Nat. Comp. 12, 101–107(2013).

[b23] Peng C. K. *et al.* Randomness versus deterministic chaos: Effect on invasion percolation clusters. Phys. Rev. A 42, 4537–4542 (1990). 9904560 10.1103/physreva.42.4537

[b24] Muller H. Fractal Scaling Models of Resonant Oscillations in Chain Systems of Harmonic Oscillators. Prog. Phys. 2, 72–76 (2009).

[b25] Dauben J. W. Georg Cantor: His Mathematics and Philosophy of the Infinite. [404] (Princeton Univ. Press, 1990).

[b26] MacLennan B. J. Universally Programmable Intelligent Matter: A Systematic Approach to Nanotechnology (presentation at IEEE-Nano, 2002).

[b27] Gurevich Y. & Shelah S. Nearly linear time. LNCS 363, 108–118 (1989).

[b28] Jozsa R. & Linden N. On the Role of Entanglement in Quantum-Computational Speed-Up. Proc. Royal Soc. London Series A. Math. Phys. Eng. Sci. 459, 2011–2032 (2003).

[b29] Maass W., Natschläger T. & Markram H. Real-time computing without stable states: a new framework for neural computation based on perturbations. Neural Comp. 14, 2531–2560 (2002). 10.1162/089976602760407955 12433288

[b30] Jean. Van H. [From Frege to Gödel: A Source Book in Mathematical Logic] in From Frege to Gödel: A Source Book in Mathematical Logic. 1879–1931 (Harvard Univ. Press, Boston, USA, 2002).

[b31] Ghosh S. *et al.* Nano Molecular-Platform: A Protocol to Write Energy Transmission Program Inside a Molecule for Bio-Inspired Supramolecular Engineering. Adv. Func. Mat. 24, 1364–1371 (2014).

[b32] Bandyopadhyay A. & Wakayama Y. Origin of negative differential resistance in molecular Junctions of Rose Bengal. Appl. Phys. Lett. 90, 023512 (2007).

[b33] Bandyopadhyay A., Sahu S., Fujita D. & Wakayama Y. A new approach to extract multiple distinct conformers and co-existing distinct electronic properties of a single molecule by point-contact method. Phys Chem. Chem. Phys. 12, 2033 (2010). 10.1039/b913691f 20165769

[b34] Sergeyev Y. D. Higher order numerical differentiation on the Infinity Computer, Optimiz. Lett. 5, 575–585 (2011).

[b35] Dirksen A. & Cola L. D. C. R. Photo-induced processes in dendrimers. Chimie 6, 873–882 (2003).

[b36] Hauer J., Buckup T. & Motzkus M. Enhancement of molecular modes by electronically resonant multipulse excitation: Further progress towards mode selective chemistry. J. Chem. Phys. 125, 061101 (2006). 10.1063/1.2243273 16942265

[b37] Ghosh S., *et al.* Resonant Oscillation Language of a Futuristic Nano-Machine-Module: Eliminating Cancer Cells & Alzheimer Aβ Plaques, Curr. Topic Med. Chem. 15, 534–541 (2015). 10.2174/1568026615666150225101155 25714385

[b38] Ghosh S. *et al.* Design and Construction of a Brain-Like Computer: A New Class of Frequency-Fractal Computing Using Wireless Communication in a Supramolecular Organic, Inorganic System. Information 5, 28–101 (2014).

