# Supplementary material for: An organic jelly made fractal logic gate with an infinite truth table
Source: Sci Rep. 2015 Jun 18;5:11265. doi: 10.1038/srep11265 (PMC4471884; doi:10.1038/srep11265)
Supplement: Supplementary Data [file srep11265-s1.pdf]

The title of all plot contains pH values and density. X axis is emission, Y axis is excitation, values of laser wavelength are in nm scale.

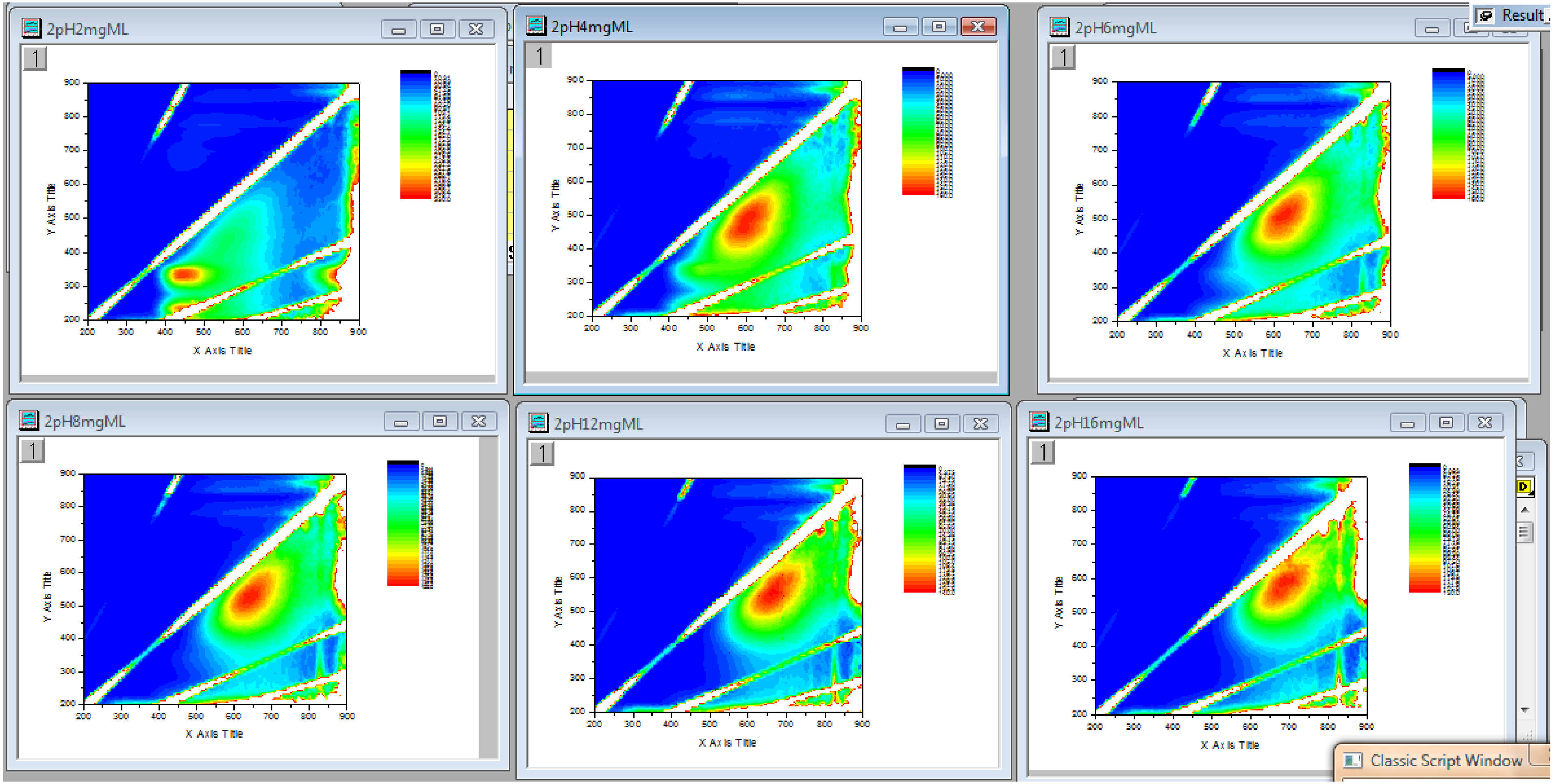

The title of all plot contains pH values and density. X axis is emission, Y axis is excitation, values of laser wavelength are in nm scale.

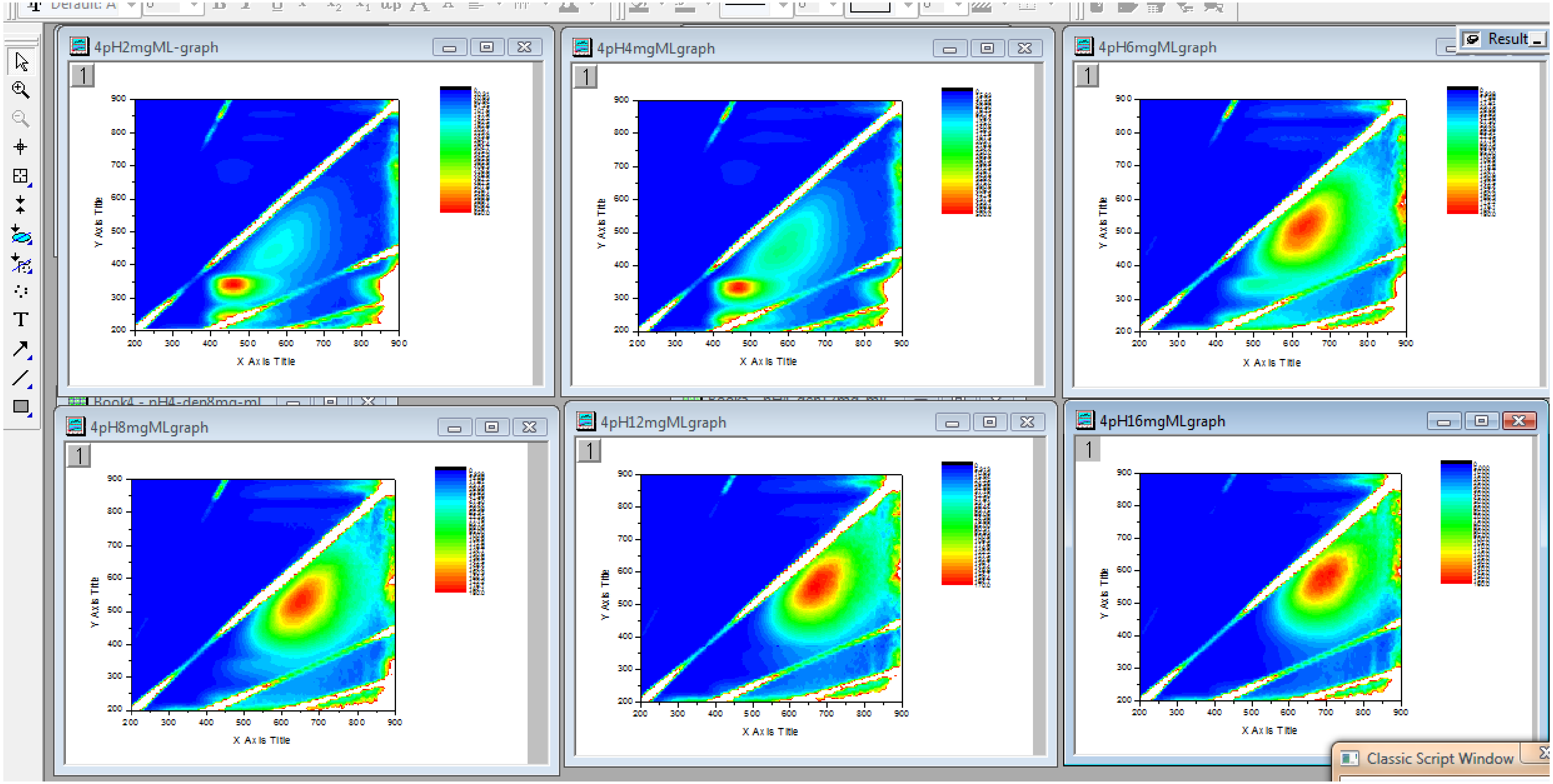

The title of all plot contains pH values and density. X axis is emission, Y axis is excitation, values of laser wavelength are in nm scale.

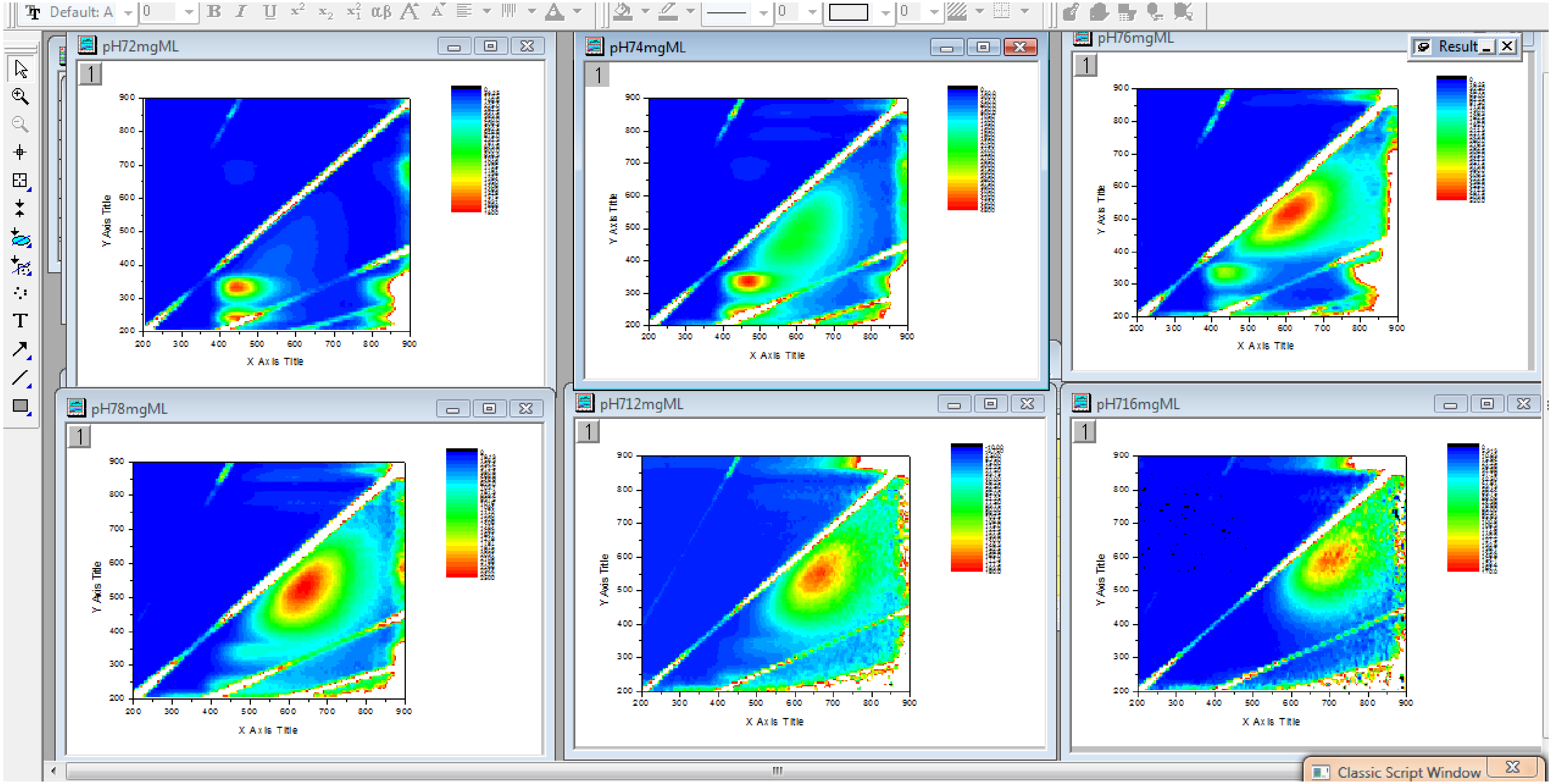

The title of all plot contains pH values and density. X axis is emission, Y axis is excitation, values of laser wavelength are in nm scale.

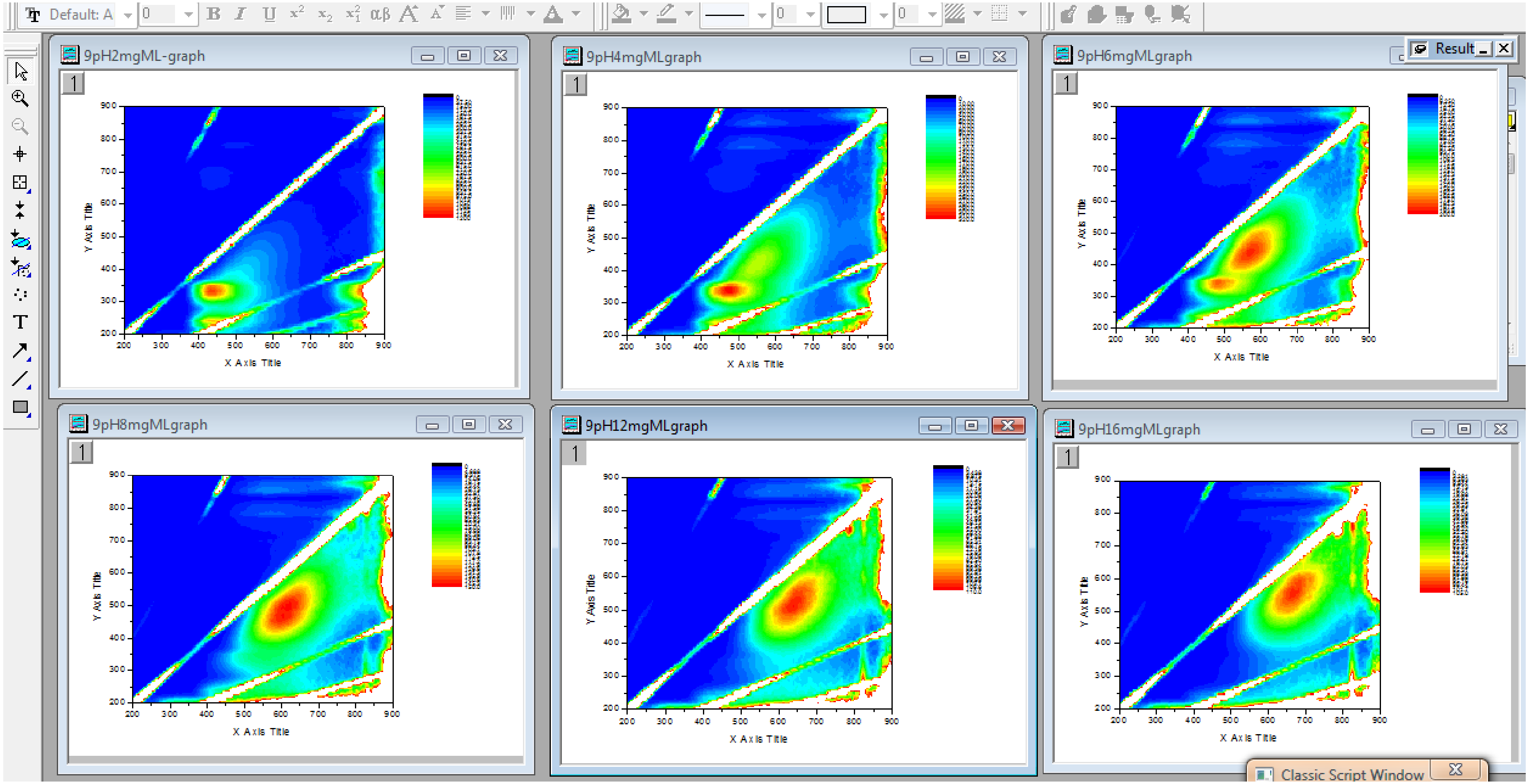

The title of all plot contains pH values and density. X axis is emission, Y axis is excitation, values of laser wavelength are in nm scale.

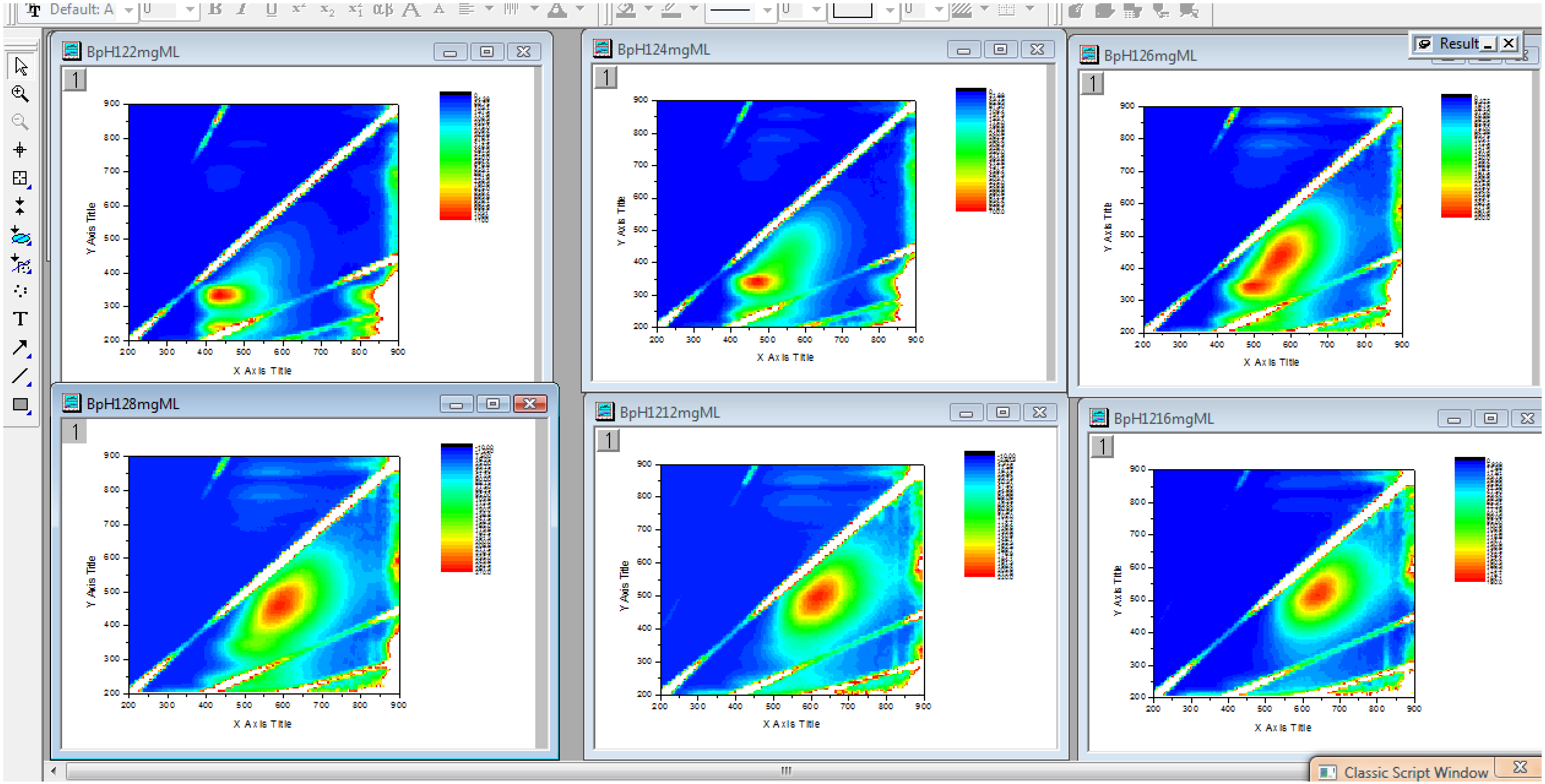

The title of all plot contains pH values and density. X axis is emission, Y axis is excitation, values of laser wavelength are in nm scale.

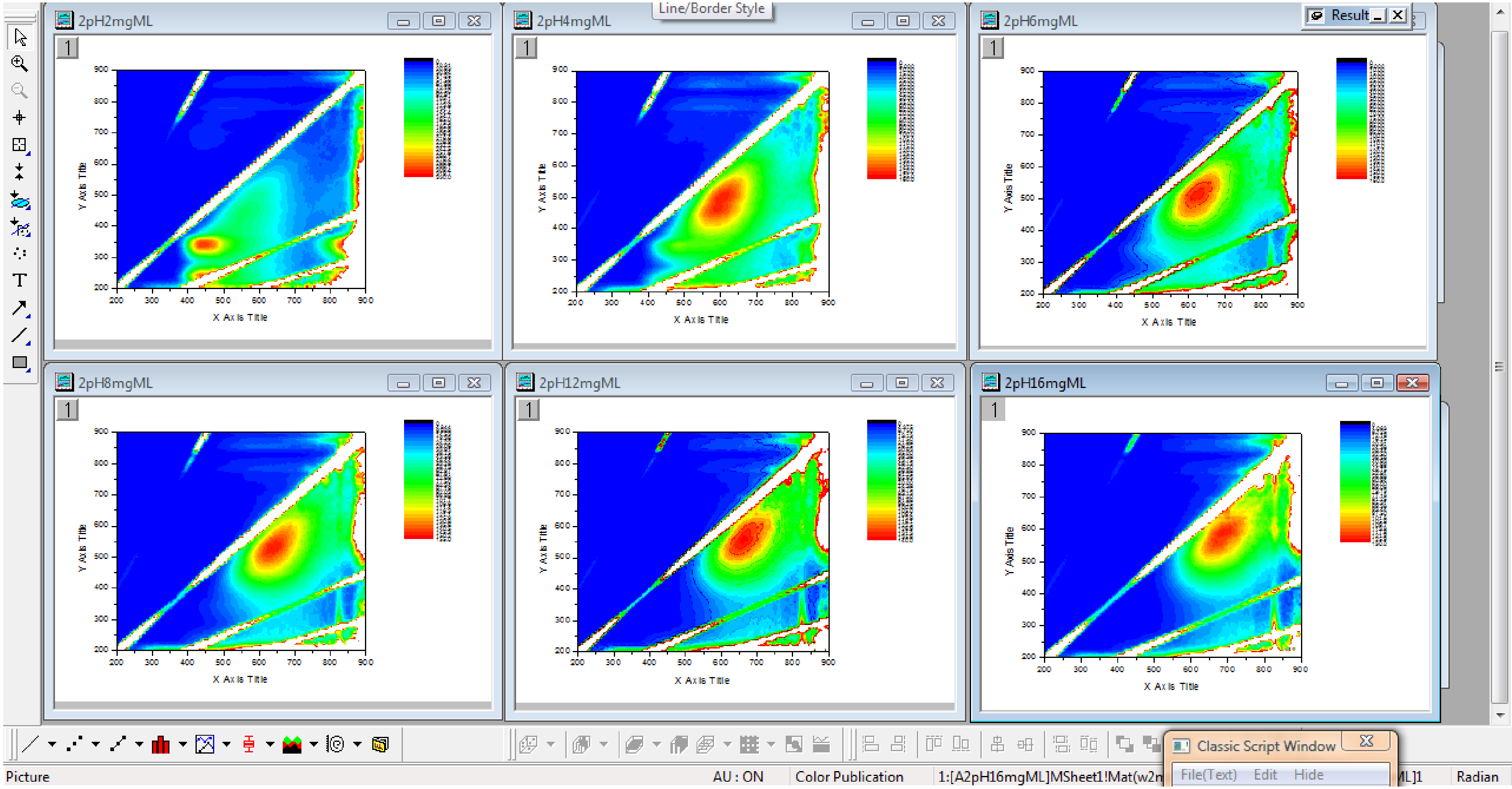

pH	2mg/mL	4mg/mL	6mg/mL	8mg/mL	12mg/mL	16mg/mL
2	Ex-3.67eV/Em-2.79eV;<br>2.79eV/2.20eV	3.56eV/2.55eV;<br>2.65eV/2.10eV;<br>2.47eV/2.04eV	2.40eV/1.98eV	2.33eV/1.93eV	2.24eV/1.88eV;<br>2.20eV/1.86eV	2.20eV/1.86eV;<br>2.10eV/1.81eV;<br>2.07eV/1.76eV
4	3.70eV/2.70eV;<br>2.81eV/2.19eV	3.71eV/2.67eV;<br>2.77eV/2.16eV	3.62eV/2.47eV;<br>2.40eV/1.97eV	2.31eV/1.92eV	2.22eV/1.85eV	2.11eV/1.81eV;<br>2.07eV/1.75eV
7	5.17eV/2.83eV;<br>3.75eV/2.81eV	3.72eV/2.63eV;<br>2.73eV/2.11eV;<br>2.46eV/2.01eV	3.57eV/2.86eV;<br>2.36eV/2.01eV	3.65eV/2.51eV;<br>2.33eV/1.93eV	2.23eV/1.84eV	2.04eV/1.72eV
9	3.72eV/2.86eV	3.63eV/2.62eV;<br>2.95eV/2.26eV	3.59eV/2.58eV;<br>2.79eV/2.17eV	2.64eV/2.06eV;<br>2.51eV/2.01eV	2.42eV/1.93eV<br>2.32eV/1.90eV	2.23eV/1.86eV
12	3.71eV/2.85eV	3.60eV/2.63eV;<br>3.0eV/2.27eV	3.55eV/2.50eV;<br>2.83eV/2.19eV	3.55eV/2.88eV;<br>2.70eV/2.11eV	2.48eV/2.0eV	2.41/1.94eV
