# Supplementary material for: An organic jelly made fractal logic gate with an infinite truth table
Source: Sci Rep. 2015 Jun 18;5:11265. doi: 10.1038/srep11265 (PMC4471884; doi:10.1038/srep11265)
Supplement: Supplementary online material [file srep11265-s8.doc]

# Supporting online material

# An organic jelly made fractal logic gate with an infinite truth table

**Subrata Ghosh^1,2^, Daisuke Fujita^1^ & Anirban Bandyopadhyay^1^**

*^1^National Institute for Materials Science (NIMS), Advanced Key Technologies Division, 1-2-1 Sengen, Tsukuba, Japan.* *^2^Mass General Hospital (Harvard Medical School), Building-114, 16th Street, Charlestown, Boston,*

*Massachusetts-02129 USA.*

(Ghosh et al)

Online Table and Text:

Table 1: The comparison between Conventional logic gate and Fractal logic gate

1. Basics of chaos and determinism:
2. What is CEES?
3. Mathematical model of linear chain generating fractal
4. pH-density experiment : The pH-density database is compiled in a separate file.
5. Synthesis of molecular machine
6. Synthesis of P, C, M and S compositions.

List of nine Figures:

Figure S1: Proton NMR data of the molecular machine; Figure S2: C^13^ NMR data of the molecular machine; Figure S3: TOF-MALDI data of the molecular machine; Figure S4: FTIR data of the molecular machine; Figure S5: pH variation with absorbance during NR doping inside PAMAM; Figure S6a: TOF-MALDI for two different variants of NR-PAMAM-MM assembly; Figure S6b: TOF-MALDI for two different variants of NR-PAMAM-MM-sensor assembly; Figure S7: Proton NMR data of NR-PAMAM (PC); Figure S8: Proton NMR data of NR-PAMAM-Sensor (PCS); Figure S9: Proton NMR data of NR-PAMAM-Sensor-MM (PCMS)

List of six Movies

Movie 1. Molecular rotor dynamics (theory). Movie 2. Molecular rotor dynamics in STM, Movie 3. Distinctly conjugated dendritic structures. Movie 4. CEES synthesis of PCMS, the triangular energy transmission path. Movie 5. Fractal logic gate evidence. Movie 6. Single PCMS molecule oscillation under STM.

Some text note on Movie 1.

Table 1.

| **Conventional logic gate (e.g. XOR, AND, NAND etc.)** | **Fractal logic gate** |
| --- | --- |
| **Truth table is finite** | Repeated zooming unravels new truth tables over and over again. |
| **Has discrete input, if input conditions do not match, the gate does not respond, so the gate ceases to operate under noise.** | Multiple ranges infinite closely spaced inputs, even if the inputs change by little, the system can find a distinct output, so it does not stop operating under noise. |
| **Resolution is finite, i.e. range of inputs and domain of the output are quantized** | Infinite resolution, any given input range could be expanded forever. It defies the measuring instrument. |
| **Conventional logic gates are not adaptive, input-output has just a relation, hence, not programmable.** | The logic gate is adaptive, with the changing environment, for the same input we get different output, hence programmable. |
| **Conventional logic gates are mostly linear, fixed mathematical functions explain input, output relation, hence “one device-one gate” is followed.** | Truth table is made of non-linear functions, in different ranges, different functions operate. A single hardware is a composition of many gates. |
| **Composition of inputs is finite (e.g. 0 0 1 for a 3 input logic gate).** | Even if we do not zoom, astronomically large number of input compositions could be created. |

1. **Chaos and determinism:**

With fractal logic gate we want to bridge the gap between chaos and determinism since chemical reactions could be both deterministic and chaotic at a time. If bridged, a chemical system could deliver the programmed output even under noise. Let's explain the chaos and determinism.

Unlike deterministic system, a chaotic system is apparently random but predictable only if we know the initial condition (e.g butterfly effect), thus one can calculate the output of such a multi-directional chemical reaction. In contrast, in a classical random system, the same reaction would give different results in two consecutive runs. The three systems are (i) “chaotic (if we know input, then predict random output)”, (ii) “random (even if you know input, cannot predict)” (iii) “deterministic” (input-output fixed).

Here we realize quasi-determinism means Say, the solution is 1, system reaches 0.999 then after some time, 0.9999999 etc, then if conditions change, then if an output is 2, the reaction system drives towards 1.99 then 1.99999 etc. PCMS auto-motion on a surface adapts distinct geometric paths depending on the number of molecules or density.

1. **What is CEES?**

***Combined excitation emission spectroscopy (CEES):***

Combined excitation emission spectroscopy (CEES): ~200 emission spectra are recorded at excitation wavelengths with 5nm intervals. The output intensities are plotted as a function of excitation and emission wavelengths, converted into energies (eV). From iso-contour plot, we detect peaks; at each peak, we get three values, excitation energy (E_x_), emission energy (E_m_) and depending on the negative or positive sign of ΔE (=E_x_ - E_m_), absorbed or emitted energy by the molecular structure during the emission process, using solution Raman & molecular dynamics we find which atomic groups use ΔE. Using this concept we evaluate band transitions for every single event, neglecting regions above Raman ridge at 45^0^ (since E_x_ < E_m_), around 45^0^, ΔE~0, there is no absorption, entire applied energy emits out.

1. **Mathematical model for generating fractal distribution in a chain of linear oscillators**

If a tape is a linear chain of oscillator then it is already shown that it would produce time fractal or rhythms. It has been shown mathematically that scaling is a fundamental property of any natural oscillation process (Muller H., Fractal Scaling Models of Resonant Oscillations in Chain Systems of Harmonic Oscillators, Prog. in Phys. 2009, 2, 72-76.). We repeat the case of harmonics or ordered factors in a network of escape time fractal tape. We take only one tape at a time. Say, we have an oscillator with a single resonance frequency f0, the oscillator will have a higher mode oscillation frequency f1 and the relation between them f1/f0 = n, now for a nested waveform network, say one waveform encapsulates 3 waveforms in it, and that continues, then first we get f1=fo, then f1=3f0, then f1=9f0, hence in general we can write fn,p=f0 n^r. In this way, the resonant frequency spectrum due to one particular symmetry can be represented as a logarithmic fractal spectra. We can clearly see that a singular waveform fractions continue to occur in the chain of oscillators. If f0 is fundamental resonance of one oscillator and f is frequency of the chain then using simple expression of continued fraction we get the resonance spectrum or a distribution of natural resonance frequencies (Terkish V. P., The continued fraction method, Leningrad, 1955), f=f0exp(S), S=n0/z + z/(n1+z/(n2+z/(n3+….+z/ni))). Now, the band we get for i=1, is similar to band we get for i=2 and so on, so it is a fractal, the spectrum looks like a hyperbolic function (H. Muller, Progress in Physics, 2, 72 (2009)).

1. **pH-density experiment: The complete database in the form of row CEES data is provided in a separate pdf file.**

The pH-density variation experiment is a simple spectroscopic measurement. The solution volume was kept constant. Synthesizing the organic products are very time consuming and yields are not as much as one can assume. Hence, a major challenge for this experiment was producing enormous amount of PCMS for the pH variation study. Always the volume was kept constant in the quartz cell. Here we provide a small data set where actually the change took place, in practice, we did study much more accurately. One such high resolution data is provided in the Movie 5.

**Some notes on Movie 1.** Though the majority of molecular dynamics data of this paper is constructed using Hyperchem, to make this video, we use freely available XenoView for Windows (Visualization for Computer Simulation), The File Version: 3.7.3.0, we acknowledge Sergei Shenogin. This software uses universal PCFF-type force fields with anharmonic corrections, tailored to study the properties related to the vibrational spectra of the material (thermal properties first of all). Note that for non-bonded interactions the actual distance parameters are r^3^ or r^6^ (cube or the 6th power of zero-force distance), depending on the force field. Since electrostatic screening and hydrophobic effects are masked by viscous drags of the solvents, and vacuum conditions are strictly inapplicable, therefore, Langevin Dynamics is suited, however, XenoView provides similar output.

Before starting the Molecular Dynamics simulation, global energy minimization was carried out on the molecular structure (pdb file) for 100 steps. Thus, we obtained the molecular car file after energy minimization, which was considered for the Molecular Dynamics simulation study. The values of the parameters set for the simulation are: Temperature 298K, Time step: 0.5 fs, number of steps: 50000, data output steps: 10, structure output steps: 500, at constant volume & shape, no thermostat & tolerance (kCal/mol) is 1.0000E-004.

**E. Synthesis protocol for the machine molecule (MM or M):**

An important note on PCMS synthesis:Why it is so different than PCM?

The synthesis reported in this manuscript is a product PCMS and the previously reported product was PCM. Both are very different in the sense that P+C=PC, then PC+S=PCS and then PCS+M=PCMS. Now, for PCM, P+C=PC and PC+M=PCM. The number of machines connected to PCM and PCMS are different and if one connects M before S, then synthesis of PCMS is impossible, thus the route to synthesis PCMS is fundamentally different in several experimental conditions aspect and should therefore not mixed. For this reason, if the reader assumes that our previous synthesis of PCM is similar to PCMS, just adding a new S, then, it would be injustice to this work.

**Step I**

2-Aminobromo naphthalene (5 g, 0.023 mol) (**I, left, starting material**) is treated with di *t*-butyl dicarbonate (4.9 g, 5.18 mL, 0.023 mol, see above arrow for the structure) in dry toluene (100 mL) and heated at 70^o^C for 15h. When TLC confirms the completion of the reaction, the reaction mixture is repeatedly washed with water, the remaining material is dried using anhydrous sodium sulfate so that the toluene is completely evaporated under vacuum.

The solid residue is re-crystallized from boiling hexane. Boc-derivative of 2-aminobromo naphthalene, (4-Bromo-naphthalen-1-yl)-carbamic acid tert-butyl ester (**II, right, the product**) was obtained as a violate color crystal, the yield of this product is 95%.

**Technique**: Boc-derivative product (**II**) is dissolved in hot hexane and filtered through the Whatman filter paper.

**Step-II**

The Boc-derivative (**II, left, starting material, above**) (3.26 g, 0.01 mol) is mixed with dry 2-ethynyl anisole (1.52 g, 0.011 mol) in dry triethyl amine (40 mL) solvent (which also acts as a base). Then the palladium acetate (catalytic amount) and triphenylphosphine (a little excess than the catalyst) are added to the mixture and heated to 100^o^C for 16h.

After completion of the reaction, triethylamine solvent is removed by keeping the mixture in vacuum and the residue is worked up with ethyl acetate. After the removal of the solvent, the resultant mixture is subjected to a column chromatography over silica gel and eluted with 10% ethyl acetate in a hexane solvent.

A white colored solid ([4-(2-Methoxy-phenylethynyl)-naphthalen-1-yl]-carbamic acid tert-butyl ester (**III, product, above, right**) is obtained, here the yield is 85%.

**Step III**

The ester (**III**) (380 mg, 1.02 mmol) is taken in dry THF (10 mL), 5 equivalent tetrabutyl ammonium fluoride (TBAF) (1.2 g, 5.1 mmol) is added and refluxed for 8h. The reaction mixture is worked with ethyl acetate after complete removal of THF. Washed with water for three times and the residue, obtained after removal of the solvent, is subjected to column chromatography. The product (**IV**) is eluted with 30% ethyl acetate in a hexane solvent. The yield is 65%.

**F. Synthesis of P, C, M and S compositions:**

Two vital points for the synthesis are that, (i) the NR or C molecules cannot be doped inside after MMs are bonded with PAMAM, doping should be done before. Moreover, (ii) the sensors are difficult to bind on PAMAM after MMs are attached. Hence, the first step is doping C; then, we attach the sensors, and finally connect the machines (MM).

The MM, C and sensor molecules are selected by optimizing synthetic possibility, non-overlapping of CEES-active regions and following several combinations, this particular set is chosen; they exhibit fluorescence in three distinct energy domains. Otherwise, identifying the role of one component is not possible.

A 5^th^ generation PAMAM dendrimer is a tree like branched shaped, colourless, solid polymer purchased in methanol solution from Aldrich. From MALDI-TOF measurement, the average mass of the dendrimer was observed to be 26000 Da which is much less than the calculated mass for 128 terminal amine groups is 28000 Da. The loss in mass is due to some structural defects resulted during the synthesis procedure and it is expected that the number of terminal amine groups are much less than estimated in the ideal structure (exactly 128 primary amine terminals). To avoid inconsistency, the dendrimer was thoroughly characterized prior use in its pristine form and since mass quality varies sample to sample, extra care was taken to have consistency during the entire synthesis process. It is highly soluble in water, ethanol, methanol, but solubility is low in DMF and DMSO and almost insoluble in other organic solvents including acetone.


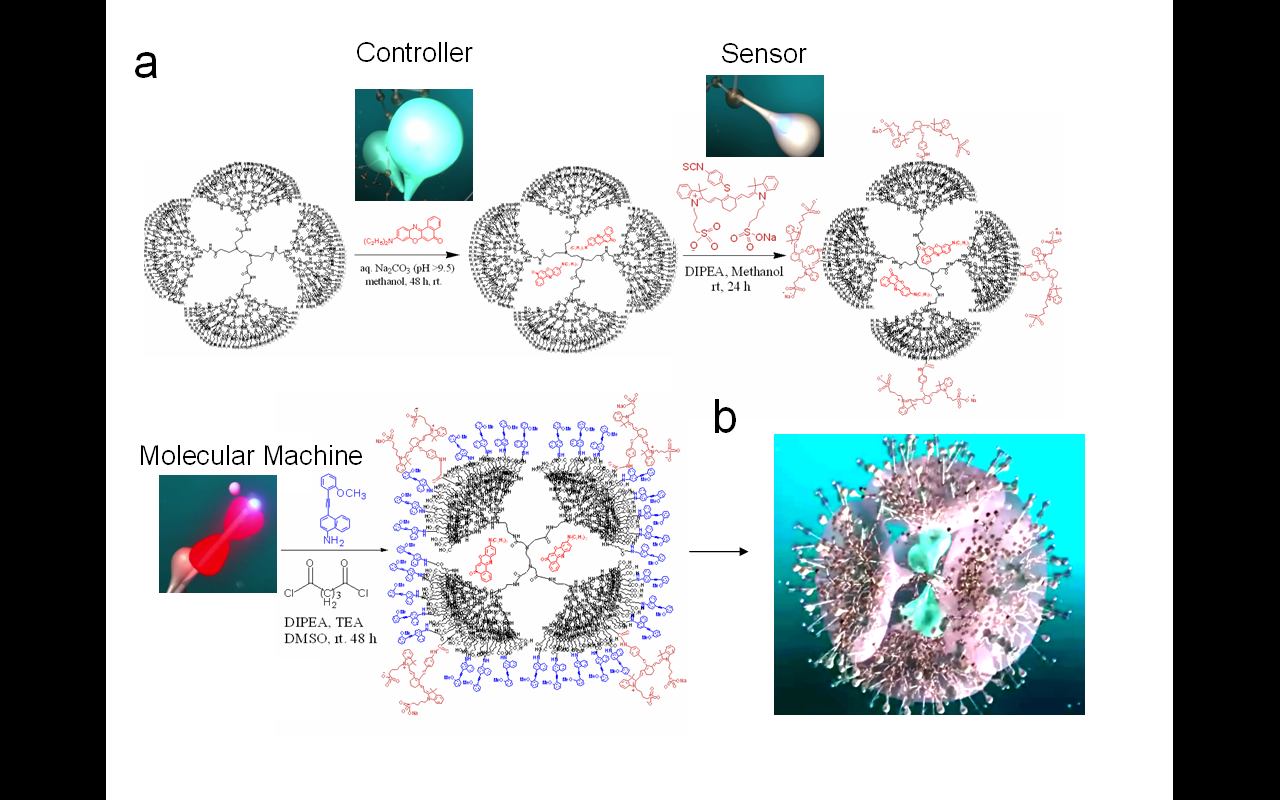


**Step-I:**

***Encapsulation of Nile-red dye molecule (C) inside PAMAM 5.0 dend. cavity (P):***

*Encapsulation of Nile-red dye molecule inside PAMAM 5.0 dendrimer cavity is highly pH dependent.*

After Changing a number of experimental conditions like different mixed solvent systems, elevated temperature, pH etc., it is found that after a particular pH 9.0 and above the Nile-red molecules to be encapsulated inside the dendritic cavities.

40 mg (0.0015 mmol, 1 mL methanol solution is pipette out) of [PAMAM G5(-NH_2_)x] dendrimer (m/z 26000 Da) is taken into a glass vial containing a magnetic bar and 1 mL methanol and 1 mL aqueous sodium carbonate (pH > 9.5) are added. The solution is stirred well and 10 mg solid crystal of Nile-red (Molecular Weight ~318.3 Da) is added in excess, the solution is allowed to stir for 2 days at room temperature (~22-25^o^C).

After 2 days of continuous stirring, the solution is concentrated by removing methanol under vacuum and the aqueous solution is filtered through Whatman filter paper, the filtrate is then dialyzed through cellulose parchment. The aqueous solution is washed with ethyl acetate and dichloromethane with mechanical shaking for several times until the organic layer becomes completely colorless that ensures the complete removal of all weakly bind Nile-red on the dendrimer surface. After removal of the solvent, the product obtained (yield 99%) is Nile-red encapsulated PAMAM dendritic box which we call as PC.

**Step-II:**

***Synthesis protocol for connecting NIR797 Isothiocyanate (S) on [NR or C encapsulated PAMAM G5 dendrimer]:***

20 mg (7.4x10^-4^ mmol) of [PAMAM G5(-NH_2_)_x_-NR] is dissolved into 3mL solution of 10%+40%+50% mixture of dimethyl sulfoxide, acetonitrile, and borate buffer containing 4 equivalent amount of dye. The reaction is stirred at room temperature for consiqutive 2 days. The acetonitrile is removed under vacuum and the reaction mixture was diluted with DI water and dialyzed for 2 days and dried to get the product PCS to use it for the next step.

**Step-III:**

***Synthesis protocol for MM attachment on {NIR797 Isothiocyanate connected [NLR encapsulated PAMAM G5 dend.]}:***

20 mg {[PAMAM G5(-NH_2_)_x_-NLR_encap_] NIR797 isothiocyanate} is added to 2 mL of dry DMSO and then we add a mixture of 1 mL of TEA (Et_3_N) and 1 mL of DIPEA (EtiPr_2_N). The mixture is stirred for 5 h then 42 mg (excess) of Molecular Machine (III) solution in DMSO (1 mL) alongwith glutaryl chloride are added and allowed to stir for 2 days at 300K, finally, the mixture is dialyzed in neutral water for 24 h to get the final product PCMS.

To confirm living cell compatibility of PCMS, first, we measured the CEES spectroscopy by applying an electric field (1V to 10V) across the supramolecular solution; we did not find any change in any of the peaks for the components. Second, we checked by changing the concentration of Na, K and other salts, yet there was no change in the response, which suggests that in the living cell routine functions will not be disrupted by PCMS.

**Figure S1**: ^1^H NMR (600 MHz, in CD_3_CN, RT) ppm: δ 8.46 (d, 1H, *J* = 8.4 Hz, Ar-H), 7.94 (d, 1H, *J* = 9.0 Hz, Ar-H), 7.62 (t, 1H, *J* = 9.0 Hz, Ar-H), 7.54-7.49 (m, 3H, Ar-H), 7.34 (t, 1H, *J* = 9.0 Hz, Ar-H), 7.05 (d, 1H, *J* = 8.4 Hz, Ar-H), 6.98 (t, 1H, *J* = 8.4 Hz, Ar-H), 6.75 (d, 1H, *J* = 9.0 Hz, Ar-H), 5.05 (s, 2H, Ar-NH_2_), 3.95 (s, 3H, Ar-OCH_3_);.

**Figure S2**: ^13^C NMR (150 MHz, in CD_3_CN, RT) ppm: δ 161.13, 146.44, 135.58, 133.85, 132.80, 130.75, 128.37, 127.86, 126.38, 124.00, 123.19, 121.91, 114.35, 112.39, 110.36, 109.36, 94.11, 89.92, 56.86.

**Figure S3**: IR (Neat): 3380 cm^-1^ (N-H), 2201 cm^-1^ (C≡C).

**Figure S4**: MALDI-TOF: m/z calcd for C_19_H_15_NO: 273.11, found [M-2H^+^]: 271.03 (calcd 271.10), [M-H^+^]: 272.06 (calcd 272.12), [M^+^]: 273.06 (calcd 273.11).

**Figure S5**: Number of encapsulated NR molecule inside PAMAM as a function of pH.

**Figure S6a**: (a) MALDI-TOF of pristine PAMAM G5. (b) MALDI-TOF of PAMAM after NR doping. MALDI-TOF of PAMAM-NR after attaching MM, the DMSO soluble part is in Figure (c) and the water soluble part is in (d). With an increasing molecular weight, discrete isolated proton peaks merge in NMR, so are the peaks of MALDI-TOF response. Ref. Lesniak et al, Bioconjugate Chemistry 2007, 18, 1148-1154; Peterson et al, European Polymer Journal 2003, 39, 33-42

**Figure S6b**: MALDI-TOF for the final PAMAM-sensor-NR-MM system, which is named as PCMS. With an increasing molecular weight, discrete isolated proton peaks merge in NMR, so are the peaks of MALDI-TOF response. Ref. Lesniak et al, Bioconjugate Chemistry 2007, 18, 1148-1154; Peterson et al, European Polymer Journal 2003, 39, 33-42

**Figure S7**: ^1^H NMR (600 MHz, in D_2_O, RT) for NR-PAMAM. With an increasing molecular weight, discrete isolated proton peaks merge in NMR, so are the peaks of MALDI-TOF response. Ref. Lesniak et al, Bioconjugate Chemistry 2007, 18, 1148-1154; Peterson et al, European Polymer Journal 2003, 39, 33-42

**Figure S8**: ^1^H NMR (600 MHz, in D_2_O, RT) for NR-PAMAM-NIR-797. With an increasing molecular weight, discrete isolated proton peaks merge in NMR, so are the peaks of MALDI-TOF response. Ref. Lesniak et al, Bioconjugate Chemistry 2007, 18, 1148-1154; Peterson et al, European Polymer Journal 2003, 39, 33-42

**Figure S9**: ^1^H NMR (600 MHz, in D_2_O, RT) for NR-PAMAM-NIR797-MM. With an increasing molecular weight, discrete isolated proton peaks merge in NMR, so are the peaks of MALDI-TOF response. Ref. Lesniak et al, Bioconjugate Chemistry 2007, 18, 1148-1154; Peterson et al, European Polymer Journal 2003, 39, 33-42
